# A Comparative Critical Analysis of Competency-Based Curricula Prescribed by Regulators for MBBS, BDS, and BSc Nursing Programs in India

**DOI:** 10.7759/cureus.89178

**Published:** 2025-07-31

**Authors:** Anupam Datta, Thivyah Prabha A. G., Nilakantan Ananthakrishnan

**Affiliations:** 1 Forensic Medicine, Agartala Government Medical College and Govind Ballabh Pant (GBP) Hospital, Agartala, IND; 2 Biochemistry, St. Peter's Medical College Hospital and Research Institute, Hosur, IND; 3 Surgery and Health Professions Education, Institute of Health Professions Education, Sri Balaji Vidyapeeth University, Puducherry, IND

**Keywords:** competency-based education, curriculum, dental, health professions education, medical, nursing

## Abstract

In Indian health professions education (HPE), competency-based education (CBE) has emerged as a transformative approach. CBE emphasizes learner-centered and outcome-driven training designed to produce clinically competent, ethically grounded, and practice-ready graduates. This comparative study critically analyses the implementation of CBE across three major health disciplines: Bachelor of Science in Nursing ((BSc Nursing), Bachelor of Medicine, Bachelor of Surgery (MBBS), and Bachelor of Dental Surgery (BDS). Through a qualitative document analysis of regulatory curricula and guidelines issued by the Indian Nursing Council (INC), National Medical Commission (NMC), and Dental Council of India (DCI), the study evaluates seven key parameters including clarity of competencies, alignment of teaching-learning methods with objectives, assessment strategies, and the integration of cognitive, psychomotor, and affective domains. BSc Nursing curriculum demonstrates the highest level of integration, clarity, and effectiveness has been observed, while all three curricula have embraced CBE in varying degrees. MBBS exhibits partial success, particularly in clinical skill development, but lacks consistency in soft skill training and assessment. The BDS curriculum remains the least aligned with CBE principles, relying heavily on traditional pedagogies. The study concludes with recommendations for interdisciplinary alignment, faculty development, and enhanced assessment systems to strengthen the implementation of CBE across all HPE in India.

## Introduction and background

Acquisition of adequate levels of competency is essential for an outgoing graduate to function effectively in the environment he is called to work after training. The effectiveness of competency-based education (CBE) in training professionals who can readily apply their knowledge and skills in practice is well-appreciated globally. The National Medical Commission (NMC), the Dental Council of India (DCI), and the Indian Nursing Council (INC) have initiated various significant reforms to their respective undergraduate (UG) curricula to move toward outcome-based frameworks. The competency-based medical education (CBME) model was introduced by the NMC for the Bachelor of Medicine, Bachelor of Surgery (MBBS) program in 2019 [1], followed by subsequent alignment efforts by the DCI (Draft Bachelor of Dental Surgery (BDS) Program Regulations 2022) [2] and the Bachelor of Science in Nursing (BSc Nursing) program by the INC in 2020 [3]. These three streams of health professions education (HPE) contribute to the major segment of healthcare professionals.

The goal of producing competent healthcare professionals is shared among the three distinct regulatory bodies, like NMC, DCI, and INC. Each council operates with a degree of autonomy, definitions of competencies, and approaches to curriculum design and assessment toward the specific needs of medical, dental, and nursing education. As yet, there is no published data on the comparison of the efficacy of the regulatory norms of CBE in these three streams. Such a comparison would enable different regulators to try and assimilate desirable points from the allied streams to improve CBE as a whole. This report aims to critically analyze the three curricula, both the recommendatory norms and the practical implementation process, on a qualitative basis and delve into these variations and their function in reality through a comparative analysis. This article will conclude with a reflective opinion of what the regulatory bodies have to learn from each other’s guidelines on UG medical education, so that the final product will be optimal for the purpose of CBE.

## Review

Methodology

A regulatory document-based comparative analysis was conducted, utilizing curriculum guidelines and related official documents published by the NMC for MBBS, the DCI for BDS, and the INC for BSc Nursing. Seven criteria were selected to provide a structured framework for the thematic analysis, allowing for a comprehensive evaluation of each curriculum's alignment with CBE principles. These criteria include (i) clarity of regulatory guidelines and objectives, (ii) concordance of listed competencies with Epstein and Hundert’s definition of competencies [4], (iii) diversity and appropriateness of teaching-learning (TL) methods for ensuring CBE, (iv) implementation of continuous monitoring and feedback systems required for CBE, (v) alignment of summative assessment with CBE evaluation principles, (vi) holistic focus on cognitive, psychomotor, and affective domains by the CBE system, and (vii) overall potential of the guidelines to produce competent health professionals who are globally employable. These are the cardinal principles of CBE and should be part of all CBE educational processes.

This methodological approach allows for a systematic and rigorous comparison of the key features and principles embedded within each curriculum framework. The whole process is focused on qualitative and pedagogical comparison of the educational process of the three major systems of HPE to enable reflection and integration into a holistic CBE system to improve the quality of the outcomes. No attempt is made for quantitative comparisons of the three streams.

Discussion

Clarity of Regulatory Guidelines and Objectives

The MBBS curriculum, under the purview of the NMC, stands out for its clearly articulated CBME framework. This framework delineates phase-wise training, specifies the roles expected of Indian Medical Graduates (IMGs), including clinician, leader, communicator, lifelong learner, professional, critical thinker, and researcher, and outlines comprehensive, outcome-focused objectives. Recent revisions in 2024 further emphasize outcome-based and patient-centric learning, alongside the integration of ethical values and communication skills through the dedicated Attitude, Ethics, and Communication module (AETCOM module). Notably, the MBBS curriculum specifies certifiable competencies for most subjects, indicating the number of times a competency must be correctly performed to achieve certification. The iterative nature of the NMC's guidelines, with revisions in 2019, 2023, and 2024, suggests a continuous process of refinement based on feedback and evolving healthcare needs. The explicit definition of the seven roles of an IMG provides a clear framework for curriculum design and the expected attributes of medical graduates [5-6].

However, despite the detailed guidelines, the sheer number of competencies, amounting to a total of 2884 to be covered in four and a half years plus one year of internship, raises concerns about feasibility [7-9]. Many of these listed competencies appear to be learning objectives rather than true, demonstrable competencies. Some are more similar to long answer questions rather than fulfilling the criteria of Epstein and Hundert [10]. Besides, the number of competencies per subject bears no relationship to the teaching hours devoted to the subject, and the certifiable competencies have no bearing on real-life requirements for many subjects. Subjects like forensic medicine, community medicine, ear, nose, and throat (ENT), general surgery, orthopedics, psychiatry, dermatology, physical medicine, radiodiagnosis, radiotherapy, and dentistry have no certifiable competencies at all (Table 1).

**Table 1:** Department-wise competencies and certifications in the MBBS curriculum MBBS: Bachelor of Medicine, Bachelor of Surgery Source: Data compiled from the National Medical Commission (NMC) Competency-Based Undergraduate Curriculum for the Indian Medical Graduate, 2019, available in the public domain and mandated by NMC for use by all medical colleges in India [7-9]

Serial number	Name of the department	Total number of competencies	Number of certifiable competencies
1	Anatomy [7]	381	2
2	Physiology [7]	127	13
3	Biochemistry [7]	69	5
4	Pharmacology [7]	85	4
5	Pathology [7]	172	3
6	Microbiology [7]	54	4
7	Forensic medicine [7]	162	0
8	Community medicine [8]	107	0
9	Ear, nose, and throat (ENT) [9]	76	0
10	Ophthalmology [9]	60	1
11	General medicine [8]	506	5
12	Respiratory medicine [8]	47	2
13	Pediatrics [8]	416	23
14	Psychiatry [8]	117	0
15	Dermatology, venereology, and leprosy (DVL) [8]	66	0
16	Physical medicine [8]	43	0
17	General surgery [9]	133	0
18	Obstetrics and gynecology [9]	126	1
19	Orthopedics [9]	39	0
20	Radiodiagnosis [9]	13	0
21	Radiotherapy [9]	16	0
22	Dentistry [9]	23	0
23	Anesthesiology [9]	46	0
Total		2884	63

In comparison, the BSc Nursing curriculum offers a well-structured, semester-based credit system that identifies 10 core competencies. The revised curriculum for BSc Nursing explicitly adopts competency-based and outcome-based approaches, integrating modular and simulation learning methodologies. The inclusion of mandatory modules within the BSc Nursing curriculum, covering areas such as first aid, health assessment, Basic Life Support (BLS), palliative care, and essential newborn care, ensures that graduates achieve a foundational level of competence in these critical areas [11]. The thrust of the Nursing curriculum is shown in Table 2. This structured approach and the emphasis on core competencies highlight the INC's commitment to providing clear guidelines and objectives for nursing education.

**Table 2:** Competencies and certification in BSc Nursing curriculum (as per Indian Nursing Council Guidelines) BSc Nursing: Bachelor of Science in Nursing; OSCEs: Objective Structured Clinical Examinations; IV: intravenous Source: Data compiled from the Indian Nursing Council Revised Regulations and Curriculum for BSc (Nursing) Program, Regulations, 2020, available in the public domain and mandated for BSc Nursing programs in India [12]

Serial number	Department/area	Core competencies/modules	Certification/assessment
1.	First aid	Emergency care, wound management, fracture handling	Mandatory module, competency assessed through clinical and practical evaluation
2.	Health assessment	Comprehensive physical and health assessment techniques	Certified through scenario-based OSCEs and supervised clinical practice
3.	Basic Life Support (BLS)	CPR, airway management, emergency response	Mandatory certification via simulation and skill checklists
4.	Palliative care	Pain management, end-of-life care, emotional support	Evaluated through reflective and scenario-based learning; clinical practice required
5.	Essential newborn care	Neonatal assessment, thermal protection, breastfeeding support	Mandatory clinical module with supervised skill practice
6.	Communication and ethics	Therapeutic communication, confidentiality, respect for diversity	Assessed via simulations, scenario-based learning, and reflective journaling
7.	Evidence-based practice	Integration of research findings into care, clinical decision-making	Included in formative and summative assessments; project-based evaluations
8.	Cultural and technological competency	Use of electronic health records, culturally appropriate care, patient safety standards	Embedded in clinical assessments and OSCEs
9.	Clinical skills and therapeutic Interventions	IV therapy, wound dressing, medication administration, vital signs monitoring	Certification through supervised clinical postings, OSCEs
10.	Leadership and collaboration	Teamwork, delegation, advocacy	Assessed through group tasks, leadership roles in practical scenarios

The BDS curriculum, however, appears to be in a more transitional phase regarding the integration of CBE principles. While the DRAFT 2022 regulations signal an intention to fully integrate CBE, the current regulations (Revised BDS Course Regulation 2007) lack the consistent level of detail that is evident in the MBBS and BSc Nursing curricula. The proposed new BDS curriculum suggests a shift toward a 4.5-year program divided into nine semesters, followed by a one-year internship, which aligns its duration with the MBBS program. This proposed change, along with the emphasis on a Learning Outcome-Based Education (LOBE) curriculum and Entrustable Professional Activities (EPAs) in the DRAFT 2022 regulations [2], indicates a move toward more clearly defined guidelines and objectives for dental education in India. This is, however, yet to be notified as mandatory regulations.

Authenticity of Competencies (as per Epstein and Hundert's Definition)

The MBBS curriculum, through its dedicated AETCOM module, demonstrates a strong alignment with Hundert’s definition of professional competence, focusing on attitude, ethics, and communication. This module, alongside the curriculum's emphasis on clinical reasoning, reflective practice, and the integration of simulation-based learning [1], indicates a commitment to developing well-rounded medical professionals. The AETCOM module specifically aims to enhance ethical values, improve responsiveness to patient needs, and foster effective communication skills among future doctors. The dedicated focus on these soft skills signifies a conscious effort to cultivate professionalism and patient-centered care from the early stages of medical education. However, the assessment of soft skills, including those covered in AETCOM, is not consistently robust, with AETCOM assessments often relying on theory-based questions rather than practical application or demonstrations of skills [13].

The BSc Nursing curriculum effectively incorporates elements aligning with Hundert's definition. It emphasizes ethics, communication, patient-centered care, and evidence-based practice throughout its framework. The competencies outlined for BSc Nursing reflect practice standards that include cultural diversity, communication technology, teamwork, collaboration, safety, quality, therapeutic interventions, and evidence-based practice. This broad approach ensures that nursing graduates are not only technically proficient but also possess the professional values and interpersonal skills essential for effective nursing practice [3].

In contrast, the BDS program has focused mainly on the acquisition of technical skills [14]. While these technical competencies are crucial for dental practice, the curriculum places less emphasis on soft skills, ethics, and communication. However, there is a growing recognition by the regulator of the importance of these nontechnical skills. The DRAFT 2022 BDS regulations aim to inculcate basic values of ethics and ethical principles in dental practice [2], and the inclusion of topics like dental jurisprudence and ethics in proposed curricula suggests a positive shift toward addressing the gap in affective domain development in dental education.

Diversity and Appropriateness of TL Methods for CBE

While CBME emphasizes student-centered learning and a variety of teaching methods such as small group discussions, case-based learning, and self-directed learning (SDL), the transition from traditional approaches has been slow in many institutions. The NMC mandates small group teaching with a student-to-faculty ratio of 10-12 UGs per small group. However, the lack of sufficient faculty in medical colleges, coupled with faculty members lacking the knowledge and experience to conduct small group teaching effectively within a large group student setting and a lack of infrastructure, poses a significant challenge to the uniform implementation of this method [15-16]. Measures such as the introduction of early clinical exposure are specifically aimed at bridging the gap between theoretical knowledge acquired in the preclinical years and its practical application in clinical settings. SDL is incorporated to cultivate habits of lifelong learning, essential for medical professionals in a rapidly evolving field. Integrated teaching approaches are utilized to support conceptual clarity by connecting different disciplines and promoting a holistic understanding of medical concepts. However, the TL methods in the MBBS curriculum do not perfectly align with the learning objectives, potentially hindering the effective acquisition of competencies [17]. For example, in biochemistry, students are expected to interpret laboratory reports and correlate biochemical findings with clinical conditions; yet, teaching is often limited to rote memorization of pathways rather than case-based discussions that build diagnostic reasoning. Additionally, practical sessions emphasize outdated manual tests with little exposure to automated analyzers, quality control processes, or the clinical integration of biochemical data, limiting the development of diagnostic and analytical skills. Furthermore, the hidden curriculum in MBBS education aims to significantly shape students into first-contact physicians beyond the attainment of specific skills, by emphasizing the need for effective integration into a system-based approach. The lack of alignment between intent and practice hinders the process of cultivating competent first-contact physicians. The use of simulation labs provides a safe environment for students to practice essential medical procedures, while Demonstration-Observation-Assist-Perform (DOAP) sessions facilitate skill acquisition [6]. Clinical clerkships offer invaluable hands-on experience in patient care. However, compressed course durations and resource constraints often limit practical exposure [18].

The BSc Nursing program utilizes a range of TL methods, including experiential and scenario-based learning, simulations, and modular teaching. The curriculum integrates blended learning approaches, incorporating reflective and simulated learning experiences, concept maps, etc. These methods are closely aligned with the expected outcomes of the program, with scenario-based learning and clinical practice specifically designed to cultivate safe and ethical nursing professionals. The BSc Nursing curriculum also exhibits a tight alignment between its prescribed TL methods and learning objectives through the use of scenario-based learning and extensive clinical practice. Scenario-based learning provides students with opportunities to apply their knowledge and skills in simulated real-world situations, thereby reinforcing their understanding and preparing them for clinical practice. The significant emphasis on clinical practice ensures that nursing students gain hands-on experience and develop the necessary competencies under the guidance of experienced mentors. This close alignment of teaching methods with learning objectives contributes to the curriculum's effectiveness in producing safe and ethical nursing professionals. The above is the reason that nurses who have completed their UG education in India find it easy to secure employment both in India and abroad. In BSc Nursing, the teaching methods effectively utilize simulations and reflective learning to shape safe and ethical nursing professionals [3]. Simulation-based learning allows students to practice a wide range of nursing skills in realistic scenarios without risk to patients. Although this happens in medicine also, it is to a far lesser extent. Reflective learning encourages students to critically analyze their experiences and develop a deeper understanding of nursing practices [19-20]. These methods are highly appropriate for achieving the expected outcomes of producing competent, caring, and ethical nurses who can provide quality care in diverse healthcare settings.

In contrast, the BDS curriculum has historically relied more heavily on traditional teaching methods such as lectures and clinical demonstrations [14]. However, initiatives are underway to integrate simulation-based learning, gamification techniques, peer-assisted learning, and various forms of technology to enrich the learning process. Furthermore, case-based learning and problem-based learning are being explored as effective strategies to foster critical thinking and clinical reasoning skills among dental students [21]. However, the movement still has to gain ground or velocity. While traditional lectures and clinical demonstrations are effective in conveying foundational knowledge and demonstrating basic dental procedures, there are identified gaps in nurturing essential competencies such as communication, professionalism, and interdisciplinary collaboration [14]. The BDS curriculum requires enhancements in its teaching methods, particularly in the training of soft skills and the development of digital literacy, to better meet contemporary professional demands [21-22]. While traditional clinical demonstrations are essential for developing procedural skills, there is a need to integrate more active learning methods that foster communication, professionalism, and interdisciplinary collaboration. Incorporating training in digital dentistry and the use of electronic health records is also crucial for preparing dental graduates for the increasingly digital healthcare environment.

Continuous Monitoring and Feedback Systems Required for CBE

The MBBS program places a significant emphasis on continuous monitoring and feedback systems through formative assessments conducted via internal assessments, the maintenance of logbooks, and the provision of structured feedback to students [23]. However, based on the faculty involvement across different institutions, the effectiveness of these systems varies. Hence, the consistency and quality of reflective feedback suggested to students may not be complete [24-25].

Integration of internal evaluations and feedback mechanisms is more systematic in the BSc Nursing curriculum compared to the BDS program [3]. To continuously monitor student learning and provide timely feedback, diverse formative assessment methods are employed, enabling early identification of learning gaps for appropriate support.

Informal and unstandardized feedback systems are mostly followed in the dental curriculum [14]. Hence, the effective monitoring of student progress can be affected and may result in inconsistent feedback experiences for students. Recognizing the importance of feedback in a CBE model, there is a need for BDS programs to develop and implement more structured and systematic approaches to continuous monitoring and feedback to ensure that students receive the guidance necessary for competency development.

It must be remembered that continuous significant feedback from formative assessment to students on their performance to promote reflective learning and the need for additional inputs for those who fail to reach the expected norms is an unavoidable mandate for CBE.

Alignment of Summative Assessments With CBE Evaluation Principles

Summative assessments in the MBBS program are increasingly reflecting a shift toward competency assessment, particularly with the forthcoming National Exit Test (NExT) [1]. Tools such as Objective Structured Clinical Examinations (OSCEs) and Objective Structured Practical Examinations (OSPEs), along with Mini-Clinical Evaluation Exercises (Mini-CEX), enhance objectivity [26]. However, OSCE and OSPE assessments are not yet standardized across all medical colleges in India [27]. Furthermore, summative assessments in theory often focus on recall-based questions with less emphasis on problem-solving [28]. Additionally, formative assessment marks are not added to the summative assessment to determine pass/fail status in MBBS, unlike in BSc Nursing. The formative examination marks are only used as a gate for allowing the student to proceed to the summative examination, provided he/she has scored a minimum of 50% cumulatively in formative assessments. They are not entered in the marksheet and do not count for pass/fail or ranking.

The BSc Nursing curriculum integrates OSCEs and clinical evaluations that are explicitly aligned with the core competencies expected of nursing graduates [3]. These assessment methods ensure that the summative evaluations measure not only theoretical knowledge but also the practical skills and professional competencies essential for effective nursing practice in various healthcare settings.

BDS assessments rely predominantly on traditional theory examinations and viva-voce formats, with a limited structured evaluation of soft skills or clinical performance [14]. However, the DRAFT 2022 BDS regulations mention the NExT exam for dental graduates [2], and there is a growing adoption of OSCEs/OSPEs in some institutions [29], indicating a future direction toward more competency-based summative assessments in dental education.

Holistic Focus on Cognitive, Psychomotor, and Affective Domains

The MBBS curriculum explicitly addresses all three domains, with the AETCOM module focused on the affective domain. Clinical exposure supports psychomotor learning, while theory caters to the cognitive domain. BSc Nursing demonstrates balanced domain coverage through practical and values-based education [3]. BDS, while strong in cognitive and psychomotor training, underrepresents the affective domain, including ethical reasoning and communication [12].

Overall Potential to Produce Competent Health Professionals Who Are Globally Employable

The MBBS curriculum exhibits strong potential to produce competent, adaptable healthcare providers, especially if implementation challenges are addressed. BSc Nursing education is highly aligned with CBE ideals, providing graduates with a well-rounded foundation in professional competencies. The BDS curriculum, while clinically focused, requires strategic reforms to meet contemporary healthcare demands.

The Way Ahead

For the MBBS curriculum under the NMC, it is recommended to streamline the number of competencies to focus on core, essential skills, ensuring that they represent true competencies rather than merely learning objectives. Assessment of soft skills should move beyond theory-based questions in the AETCOM module to include practical scenarios and direct observation. There is a pressing need to standardize OSCE and OSPE assessments across all medical colleges to ensure consistency and reliability in evaluating clinical skills. The implementation of reflective feedback mechanisms should be enhanced to better support student learning and professional development. Strengthening guidance and mentorship within the learner-doctor method is vital to ensure effective student support and supervision. Incorporating more problem-solving questions into summative theory examinations would facilitate the evaluation of critical thinking and application of knowledge. To have a comprehensive assessment of clinical competencies, as in the BSc Nursing curriculum, the feasibility of integrated OSCEs should be explored. A holistic view of student performance can be achieved by including formative assessment marks in summative evaluations, as practiced in BSc Nursing. Through focused faculty training and clear guidelines, the implementation of SDL must be standardized across all medical institutions.

Additionally, there is a need to focus on interactive teaching and small-group facilitation in faculty development programs in order to shift away from traditional lecture-based methodologies to CBME. An alignment between TL methods and learning objectives has to be achieved to enhance competency acquisition. There is also a need in all three streams to use a diverse set of workplace-based assessments tailored to measure the competencies that the teachers are striving to measure.

Simulation-based learning and interprofessional education have to be introduced in the BDS curriculum under the DCI. Ethical aspects, communication, and professionalism should be integrated into the curriculum. To enhance the clinical competence evaluation, integrated OSCEs and structured feedback mechanisms should be adopted. Ethical education, reflective practices, and simulation-based learning have to be consolidated in the BSc Nursing curriculum. The consistency of SDL implementation and clinical mentorship has to be improved. In order to meet the evolving healthcare roles among the nursing students, it is the need of the hour to foster research literacy and leadership development among them.

At the cross-disciplinary level, interprofessional education should be actively promoted across the MBBS, BDS, and BSc Nursing programs to encourage collaborative practice and teamwork. Developing common assessment rubrics aligned with the CBE principles and ensuring periodic curriculum updates to reflect emerging healthcare challenges are crucial for maintaining relevance and quality in HPE. The differences in approach between the three streams are shown in Table 3.

**Table 3:** Comparative analysis of MBBS, BSc Nursing, and BDS curricula MBBS: Bachelor of Medicine, Bachelor of Surgery; BSc Nursing: Bachelor of Science in Nursing; BDS: Bachelor of Dental Surgery; AETCOM: Attitude, Ethics, and Communication; IMG: Indian Medical Graduate; CBE: competency-based education; CBME: competency-based medical education; T/L: teaching-learning; DOAP: Demonstration-Observation-Assist-Perform; FA: formative assessment; EBP: evidence-based practice; NExT: National Exit Test; OSCE: Objective Structured Clinical Examination; OSPE: Objective Structured Practical Examination; Mini-CEX: Mini-Clinical Evaluation Exercises

Serial number	Attributes of the curriculum being compared	Medical (MBBS)	Nursing (BSc Nursing)	Dental (BDS)
1	Clarity of regulatory guidelines	Clear, detailed CBME framework, but an overwhelming number of competencies	Clear guidelines with a structured credit-based system	Still evolving; 2022 draft suggests improvements
2	Well-defined outcomes	Well-defined, phase-wise with IMG roles and AETCOM integration	Clearly identified 10 core competencies	Not well defined in existing regulations; draft provides direction
3	Authenticity of competencies listed	Partially authentic; some are learning objectives or exam-style questions	Authentic; based on ethics, communication, teamwork, EBP	Primarily technical competencies; limited soft skill focus
4	Alignment of T/L methods with CBE	Moderate; intended but poorly aligned due to resource and training gaps	High alignment: scenario-based, blended, modular, reflective methods	Traditional methods dominate; reforms underway
5	Early clinical exposure	Present as part of curriculum	Extensive early practical exposure	Mentioned but not emphasized historically
6	Clinical clerkship or equivalent	Included but limited due to time and faculty constraints	Strong clinical exposure embedded in training	Present, traditional clinical postings dominate
7	System of continuous FA and feedback	Mandated; logbooks, IA, but quality varies across institutions	Systematic formative assessment and feedback mechanisms	Informal; lacks structured feedback and monitoring
8	Weightage of FA in summative process	Formative assessment only gatekeeper, not counted for pass/fail	Formative marks counted in the summative exam	Not integrated with summative exams
9	Alignment of summative examination with principles of CBE to ensure desired outcome	Upcoming NExT, OSCE/OSPE/Mini-CEX used but non-standardized	OSCEs, clinical evaluations clearly mapped to core competencies	Still traditional; some colleges use OSCE/OSPE; draft suggests NEXT
10	Focus on the cognitive domain	Strong theoretical component	Well balanced with theory and reflective learning	Focused mainly on theory in dental sciences
11	Focus on skill development	Strong in clinical subjects; uses DOAP and simulation	High practical exposure, aligned with skill domains	Strong technical training, limited simulation use
12	Focus on soft skills and professionalism	AETCOM exists; soft skill assessment is weak and theory-based	Strong emphasis on ethics, professionalism, and communication	Low current focus; draft includes ethics/jurisprudence

## Conclusions

This review suggests that there is a gap between the intended curriculum, the taught curriculum, and the learnt curriculum in all three streams of HPE in India. Among India’s healthcare education programs, the MBBS curriculum, when implemented effectively, demonstrates robust alignment with CBE principles and has the potential to produce clinically competent and ethically grounded professionals. However, several areas need refinement, including the number and nature of competencies, soft skill assessment, standardization of OSCE/OSPE, feedback mechanisms, mentorship, the focus of summative assessments, the prevalence of teacher-centric teaching, challenges in small group teaching, alignment of TL methods with objectives, the impact of the hidden curriculum, and the need for better integration. The BSc Nursing curriculum offers a balanced, well-integrated model, particularly strong in addressing professional and affective domains. The BDS curriculum, though evolving, requires more rapid and comprehensive reform. Coordinated efforts in faculty development, curricular integration, and assessment reform are essential for all three programs to fully realize the promise of CBE in India's healthcare landscape.
